# Transcriptomic Study on Human Skin Samples: Identification of Two Subclasses of Actinic Keratoses

**DOI:** 10.3390/ijms24065937

**Published:** 2023-03-21

**Authors:** Hélène Dubois-Pot-Schneider, Grégoire Khairallah, Cyril Brzenczek, François Plénat, Frédéric Marchal, Marine Amouroux

**Affiliations:** 1 Université de Lorraine, CNRS, CRAN, 54000 Nancy, France; 2 Department of Plastic, Aesthetic and Reconstructive Surgery, Metz-Thionville Regional Hospital, 57530 Ars-Laquenexy, France; 3 Département de Chirurgie, Institut de Cancérologie de Lorraine, 54519 Vandœuvre-lès-Nancy, France

**Keywords:** human skin, squamous cell carcinoma, actinic keratosis, transcriptome, classification

## Abstract

Actinic keratoses (AKs) are sun-damaged skin areas that affect 20% of the European adult population and more than 50% of people aged 70 years and over. There are currently no clinical or histological features allowing us to identify to which clinical class (i.e., regression or progression) an AK belongs. A transcriptomic approach seems to be a robust tool for AK characterization, but there is a need for additional studies, including more patients and elucidating the molecular signature of an AK. In this context, the present study, including the largest number of patients to date, is the first aiming at identifying biological features to objectively distinguish different AK signatures. We highlight two distinct molecular profiles: AKs featuring a molecular profile similar to squamous cell carcinomas (SCCs), which are called “lesional AKs” (AK_Ls), and AKs featuring a molecular profile similar to normal skin tissue, which are called “non-lesional AKs” (AK_NLs). The molecular profiles of both AK subclasses were studied, and 316 differentially expressed genes (DEGs) were identified between the two classes. The 103 upregulated genes in AK_L were related to the inflammatory response. Interestingly, downregulated genes were associated with keratinization. Finally, based on a connectivity map approach, our data highlight that the VEGF pathway could be a promising therapeutic target for high-risk lesions.

## 1. Introduction

Actinic keratoses (AKs) are sun-damaged skin areas developed predominantly on sun-exposed areas, particularly the forearm, hands, face, ears, bald scalp and lower legs. Featuring an increasing prevalence, AKs are extremely common and affect millions of fair-skinned people worldwide, leading to a high economic burden related to their treatment [1,2]. In European countries, the prevalence is as high as 20% of the adult population and higher than 50% for people aged 70 years and over [3]. Australia presents the highest prevalence, with 40% of the whole population affected by AKs [4,5]. AKs may follow two different clinical pathways: some regress spontaneously (or persist) and others progress towards in situ or invasive squamous cell carcinomas (SCCs). Every year, 0.6% of AKs are estimated to evolve into SCCs [6]. Even with a low individual rate of progression, most patients show multiple AK lesions leading to a 14% cumulative probability of developing an SCC within 5 years [7]. There are currently no clinical or histological features allowing us to identify to which clinical class (i.e., regression or progression) an AK belongs. For this reason, European guidelines recommend treating all AKs (or the corresponding cancerization field) [8]. In addition, the economic burden and unnecessary risk of infection, as well as the avoidable psychological impact related to surgical scars on visible (sun-exposed) body areas, should be considered [9].

AK diagnosis is typically made via clinical evaluation: AKs that appear suspicious are biopsied or even surgically removed for histopathologic confirmation. The cardinal histopathological feature of an AK is epithelial dysplasia. Roewert-Huber et al. introduced a histological classification scheme that is now commonly used for grading AK lesions [10]. The scale is based on the extent of atypical keratinocytes in the epidermis. In grade AK I (mild), atypical keratinocytes are restricted to the basal and suprabasal layers and limited to the lower third of the epidermis. In grade AK II (moderate), atypical keratinocytes extend to the lower two-thirds of the epidermis. In grade AK III (severe), full-thickness atypia of the epidermis is found and is equivalent to lesions, also called in situ SCC [9,11].

Although AK I lesions were initially considered “low risk”, recent studies have highlighted that it is not possible to predict which AK will progress, regardless of the grade. Indeed Fernandes-Figueras et al. showed that AK I lesions are the most common precursors of SCC, arising through the “differentiated pathway” [12]. Moreover, clinical classifications do not accurately match histology [13].

In this context, the identification of biomarkers that could help to stratify AK risk and potentially predict the fate of each lesion is becoming increasingly important. In 2013, Hameetman et al. performed unsupervised transcriptomic profiling on individual lesions and revealed that the gene expression profiles of AK are heterogeneous [14]. Based on their molecular profile, some AKs are similar to SCCs while few AKs are similar to normal tissue. More recently, Chittsazzadeh et al. confirmed that even if a uniform histological criterion is used, there is a spectrum of AKs that, on the transcriptional level, resemble normal tissue versus some that resemble SCCs [15]. The transcriptomic approach seems to be a robust tool for AK characterization, but there is a need for additional studies including more patients and elucidating the molecular signature of AKs. In this context, the present study, including the largest number of patients to date, is the first aimed at identifying biological features to objectively distinguish different AK signatures. Two distinct molecular profiles were highlighted: AKs featuring a molecular profile similar to SCCs and AKs featuring a molecular profile similar to normal skin tissue. We suggest the hypothesis that each corresponds to a higher- or lower-risk class of evolution, respectively.

## 2. Results

### 2.1. Principal Component Analysis (PCA) Based on Differentially Expressed Genes (DEGs)

To determine the optimal pipeline for analyzing the transcriptomic data, a cohort of reference samples was labeled according to the histological diagnosis: 15 NL (NL corresponds to the healthy (H) histological class) and 17 L (L corresponds to the SCC histological class) (Table S1, Figures S1 and S2).

**Figure 1:**
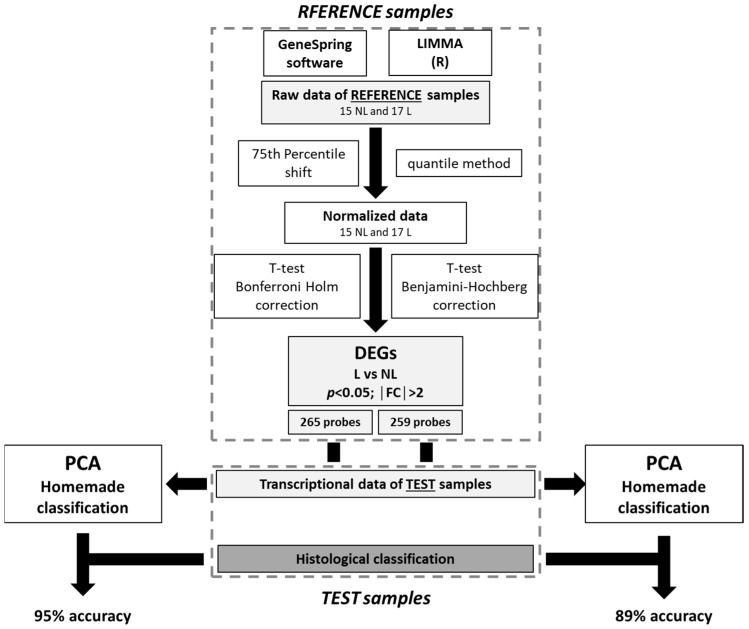
Microarray data analysis pipeline selection.

Two microarray analysis pipelines were used to determine significant DEGs (Figure 1).

The DEGs obtained from the LIMMA pipeline and GeneSpring software using the samples from the reference group were composed of 265 and 259 transcripts, respectively (Table S2).

The DEGs obtained were then used to produce two PCAs. The samples in the test group, first labeled “L” or “NL” according to the histological analysis, were placed in the space of each PCA presented in Figure S3. Both PCAs allowed us to qualitatively visualize a difference in the expression between the L and NL samples. The PCAs revealed a greater variability in expression among the L samples than the NL samples, with the latter appearing to be more clustered on the plane of the two PCAs.

### 2.2. Homemade Classification

The histological label and the homemade classification label relative to the centroid distance of the test group for both differential analysis methods were compared. The histological labeling was considered the ground truth, and the homemade classification using the LIMMA DEGs showed an 89% accuracy, with two misclassified samples, whereas the homemade classification using the GeneSpring DEGs showed a 95% accuracy with only one misclassified sample. All distances to the centroid and homemade classifications are presented in Table S3.

Based on the accuracy results, the GeneSpring DEGs pipeline was selected for the next molecular analysis and labeling of the AK samples.

### 2.3. Molecular Profiling of SCC Lesions

**Figure 2:**
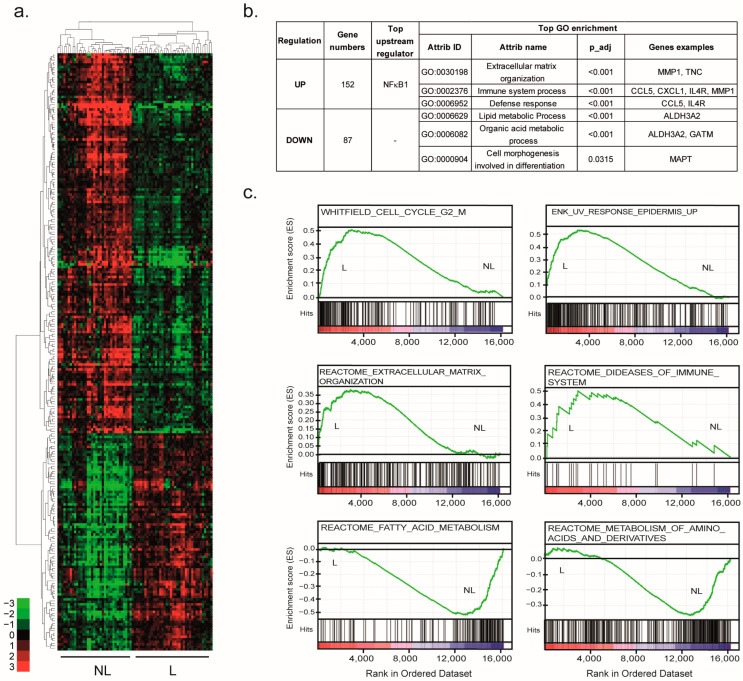
Molecular signature of SCCs. (**a**) Unsupervised hierarchical clustering of the reference samples (17 NL and 15 L) based on the DEGs selected by the GeneSpring pipeline. The cluster was visualized by Treeview. Green indicates a lower expression, and red indicates a higher expression. (**b**) The gene numbers, top upstream regulator and top GO associated with each gene group are summarized in the table. (**c**) The GSEA analysis using the gene expression profiles of reference LN samples and reference L samples. The GSEA revealed an enrichment of the “REACTOME_DISEASES_OF_IMMUNE_SYSTEM”, “REACTOME_OF_ECM_ORGANIZATION”, “WHITFIELD_CELL_CYCLE_G2_M”, “ENK_UV_RESPONSE_EPIDERMIS_UP”, “REACTOME_FATTY_ACID_METABOLISM” and the “REACTOME_METABOLISM_OF_AMINO_ACIDS_AND_DERIVATIVES” signatures (FDR < 0.25, *p* < 0.01).

The 259 DEGs (*p* < 0.05, FC > 2) identified were assigned to 239 unique gene symbols between the NL and L samples (Table S4). The subsequent unsupervised hierarchical clustering of these 239 DEGs revealed two distinct groups, separating the NL and L samples (Figure 2a). The upper part of the dendrogram grouped 152 genes upregulated in the L samples. These genes are mainly involved in the organization of the extracellular matrix (ECM) and immune response (Figure 2b and Table S5). Among these genes, there were genes encoding matrix metallopeptidase (MMP), such as *MMP1* and *MMP3*, ECM protein Tenascin C (*TNC*), or the gene encoding the alpha chain of interleukin 4 receptor (*IL4R*). The top upstream regulator of these DEGs was the Nuclear Factor Kappa B Subunit 1 (NFkB1).

The lower part of the dendrogram grouped 87 downregulated genes in the L samples related to lipid and organic acid metabolism pathways, including amino acid metabolism (Figure 2b and Table S6). In agreement, the aldehyde dehydrogenase 3 family member A2 (*ALDH3A2*) or glycine amidinotransferase (*GAMT*) was downregulated in the L samples. Then, some genes related to cell morphogenesis involved in differentiation were also downregulated in lesioned tissues, such as microtubule-associated protein tau (*MAPT*). No significant upstream regulator (FDR < 0.1) was highlighted.

In agreement, an unsupervised gene set enrichment analysis (GSEA) identified significant enrichment in the L samples (*p* < 0.01) of signatures related to immune and ECM organization, such as the REACTOME_DISEASES_OF_IMMUNE_SYSTEM and the REACTOME_OF_ECM_ORGANIZATION, respectively. Interestingly, GSEA revealed the enrichment of a high number of signatures related to the cell cycle, especially the G2/M transition, as suggested by the WHITFIELD_CELL_CYCLE_G2_M or UV response, such as the ENK_UV_RESPONSE_EPIDERMIS_UP. Interestingly, most of the gene signatures that were enriched in the NL samples were mainly related to lipid and amino acid metabolism, such as the REACTOME_FATTY_ACID_METABOLISM or REACTOME_METABOLISM_OF_AMINO_ACIDS_AND_DERIVATIVES (Figure 2c).

These results were consistent with the molecular features of the SCC transformation and, therefore, validated the transcriptomic SCC signature.

### 2.4. Identification of Two AK Classes

**Figure 3:**
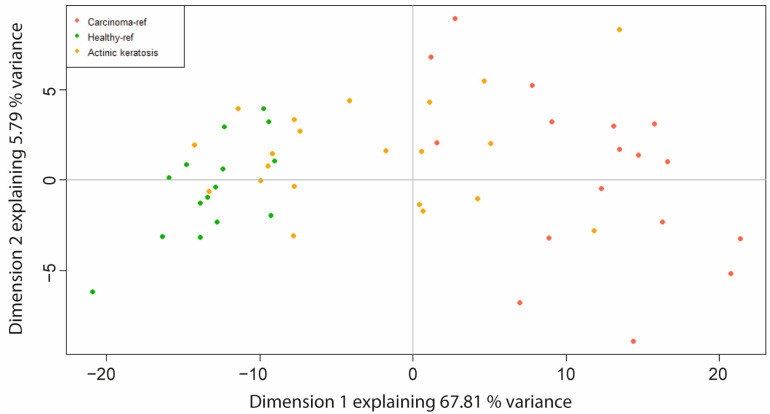
Highlighting of the two AK classes. Principal component analysis of the reference samples (15 NL “Healthy_ref” and 17 L “Carcinoma_ref”) and AK “actinic keratosis” samples (21) based on the DEGs selected by the GeneSpring pipeline.

In order to decipher the behavior of the 21 AKs collected in our study, a PCA based on the SCC signature was performed. These 259 DEGs qualitatively separated the NL from the L samples in two well-defined clusters. The NL samples demonstrated great similarities. More variations were observed within the L samples, reflecting the well-known heterogeneity of these lesions compared to the NL samples. Interestingly, the biggest transcriptomic heterogeneity was observed with the AK lesions. Indeed, the 259 DEG profiles of some of the AK samples overlapped with those of the NL samples, while others overlapped with the L samples (Figure 3).

These data demonstrate that AKs are heterogeneous preneoplastic lesions, although they are considered a unique histological class. AKs were separated into two classes by computing the distance between each AK sample and the centroid of the NL group and the L group, respectively (Table 1). Figures S4 and S5 display the histological characteristic pictures of AK_NL and AK_L, respectively.

Among the 21 AKs, 12 have a shorter distance to the NL tissue and are renamed AK_NL, and 9 have a shorter distance to the L tissue and are renamed AK_L.

**Figure 4:**
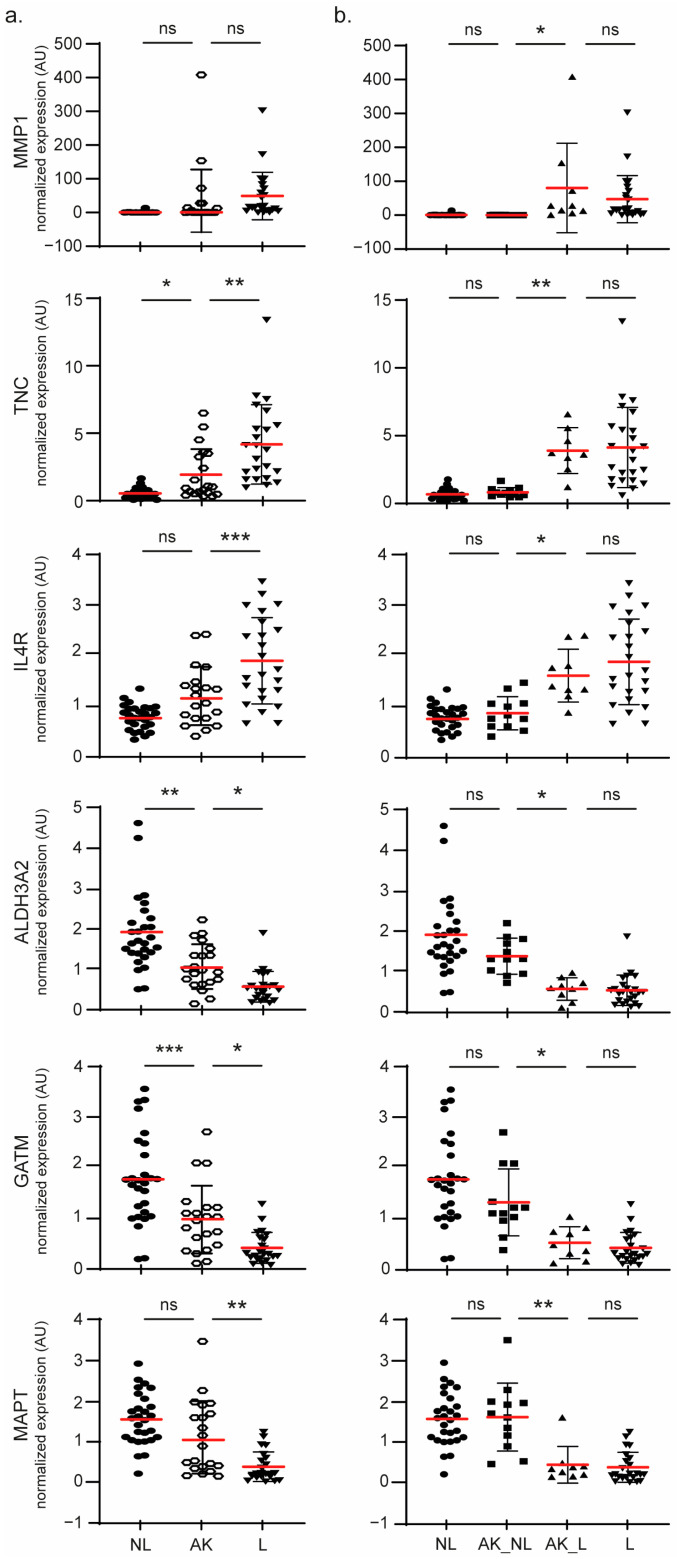
Robustness of the two AK classes’ stratification. A scatter plot showing the normalized expression for the 4 samples classes: NL (28), L (23) and AK defined as either one unique class (21) (**a**) or two different classes AK_NL (12) and AK_L (9) (**b**) for the selected DEGs. The results are expressed as the means (red lines) ± SD. The significance was evaluated by ANOVA followed by a post-hoc Tukey’s for multiple comparisons. * *p* < 0.05, ** *p* < 0.01 and *** *p* < 0.005. ns: not significant.

For the markers belonging to the SCC signature, a progressive induction or repression along the disease stages, NL, AK and L, was observed (Figure 4a).

Interestingly, when the AKs were stratified into two classes based on their transcriptomic profile, we observed that, in most cases, a significant difference in gene expression was found between the two AK classes (Figure 4b). This observation was performed with the markers involved in the most highlighted functions mentioned above. Indeed, the expression of these markers is not significantly different between the NL and AK_NL group or the L and AK_L group. Conversely, their expression is significantly decreased or increased between both new AK subclasses.

### 2.5. Molecular Signature Involved in AK Progression

To further characterize both AK classes, the molecular profile of AK_L vs. AK_NL was compared: 316 DEGs (*p* < 0.05; FC > 2) were identified using the GeneSpring pipeline described above (Table S7).

**Figure 5:**
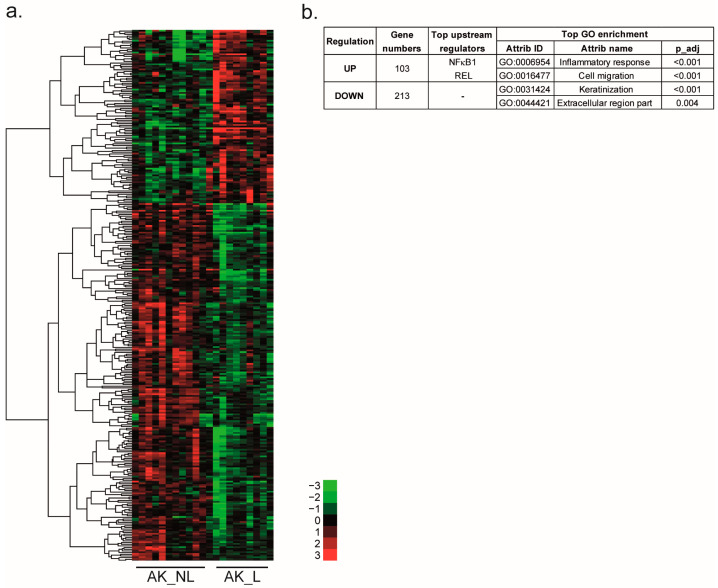
Molecular signatures of the two AK classes. (**a**) Supervised hierarchical clustering of the AK samples (12 AK_NL and 9 AK_L) based on 316 DEGs selected by a *t*-test (*p* < 0.05, FC > 2). Clustering was visualized by Treeview. Green indicates a lower expression, and red indicates a higher expression. (**b**) Gene numbers, top upstream regulators and top GO terms associated with each gene group are summarized in the table.

Among the 316 DEGs, 25% were previously observed in the SCC signature, such as *ALDH3A2*, *GATM*, *MMP1,* or TNC (Section 2.3). These common genes are highlighted in Table S7. In addition, the 237 newly identified involved a high number of genes coding cytokeratins (*KRT*) and late cornified envelope (*LCE*) protein. The supervised hierarchical clustering of the 316 DEGs separated the up- and down-regulated genes (Figure 5).

The two top enriched GO terms for the 213 downregulated genes were “keratinization” and “extracellular region part”. The 108 upregulated genes were involved in the inflammatory response and cell migration/adhesion. Upstream regulators were NFκB1 and REL (REL Proto-Oncogene, NF-κB Subunit). A connectivity map approach was used to identify molecules that could reverse the global gene expression profile induced during AK progression. One perturbagen, tivozanib, a vascular endothelial growth factor receptor (VEGFR) inhibitor, had a connectivity score lower than −90.

## 3. Discussion

Recent transcriptomic studies highlighted that even if a uniform histological criterion is used to diagnose AKs, there is a spectrum of AKs that, on the transcriptional level, resemble normal tissue versus some that resemble SCCs [15,16,17,18]. However, the low number of AK samples in these studies did not allow us to distinguish AK subclasses based on their transcriptional profile. To date, there are no AK patient cohorts available in the public databases. Previously, to increase the number of patient samples, integrative transcriptomic analyses were carried out [17,19]. Although these studies highlighted new pathways of, and actors in, the transformation of the SCC, they did not attempt to characterize the AK. They confirmed that their gene expression profiles show significant overlap with both normal skin and SCC tumors, as previously reported [15].

In this context, the present study, including the largest number of AK samples to date, is the first aimed at identifying biological features to objectively distinguish different AK subclasses.

Our strategy was first to identify a robust signature of SCC features. Several lists of DEGs (SCC vs. healthy tissue) were previously published. However, the methods for obtaining these lists are diverse, and because of the small sample sizes in each study, no consensus list was established. By testing two DEG selection pipelines and correlating the obtained signatures with histological classification, our study provides the most robust SCC signature to date.

Indeed, using this approach 239 DEGs in SCC compared to healthy skin were highlighted. Among them, we found six of the seven hub genes of SCC identified by Chen et al. [19]: *ARHGEF26* (rho guanine nucleotide exchange factor 26), *GATA3* (GATA-binding protein 3), *GATM*, *MMP1*, *POU2F3* (POU class 2 homeobox 3) and *PTHLH* (parathyroid hormone-like hormone). In the same way, the functional annotation of the 152 up- and 87 down-regulated genes in SCC from our study identified the enrichment of functions and pathways mainly associated with ECM organization, immune response, metabolism process, or differentiation [20,21,22,23]. The ECM plays an important role in tumor progression by regulating many functions, including proliferation, adhesion and migration, and regulates cell differentiation. Among the ECM organization signatures, we highlighted MMPs, proteases involved in ECM degradation [24]. Among them, MMP1, overexpressed in many cancers, was recently identified as a novel diagnostic and prognostic biomarker in head and neck SCC (NHSCC) [25]. In SCC, MMP1 is the most common upregulated marker found in previous studies [15,17,19,20,26,27]. Recently, Zheng et al., by comparing multiple datasets, confirmed that MMP1 may be pivotal to the transition from healthy tissue to premalignant lesions to SCC [28]. An increase in TNC, an ECM protein, which was previously shown to be increased during skin cancer development, was also highlighted [29]. Finally, in accordance with Das Mahapatra et al., the upregulation of genes involved in the immune response, such as the chemokines *CCL5* (C-C motif chemokine ligand 5) or *CXCL1* (C-X-C motif chemokine ligand 1) or *IL4R* were observed [20]. The tight association between SCC risk and immunosuppression stresses the pivotal role of the immune system in regulating SCC development and progression [30].

Such results confirm that the SCC transcriptional signature brought by our study is relevant, as genes and functional annotations highlighted in the current study were already identified in previous studies.

Based on this signature, two distinct molecular profiles of AKs were identified: 9 AKs featuring a molecular profile similar to SCCs (renamed AK_L) and 12 AKs featuring a molecular profile similar to normal skin tissue (renamed AK_NL). It can be noticed that the division of the AK class into two subclasses results from the unsupervised classification. Interestingly, this result matches the two clinical classes observed: AKs that evolve into SCC versus AKs that spontaneously regress [31]. Interestingly, 44% of the nine AK_L were diagnosed as grade I AK (AK I) by histopathology. These data confirm that AK I lesions should not be considered “low risk” and are the most common precursors of invasive SCC [12]. Our finding reinforces the hypothesis that it is currently not possible to predict which AK will progress based on histological classification, independently of its grade; the progression potential of such lesions should be investigated.

In this context, the molecular profiles of both AK subclasses were studied. A total of 316 DEGs between AK_L and AK_NL were identified. Among them, 25% of the genes were already identified in the SCC signature and were mainly related to ECM organization (*MMP1* and *TNC*) or metabolic processes (*GATM* and *ALDH3A2*). A significant difference in gene expression was clearly observed between the two AK classes. These observations confirm our hypothesis that AKs cannot be considered a single class. Our hypothesis is that expression of these markers could be a diagnostic tool to stratify lesions according to their risk of progression. Moreover, for the first time, our approach aimed at characterizing AK lesions by identifying DEGs in both AK subtypes. As for the SCC signature, the 103 upregulated genes in AK_L were related to the inflammatory response. Interestingly, downregulated genes were associated with the keratinization process as assessed by the high number of downregulated genes coding KRT and LCE proteins in AK_L. These genes are essential for epidermal differentiation. Hudson et al. already highlighted that most changes in expression levels in SCC compared to normal tissue were related to epidermal differentiation. In AK_L, the downregulation of multiple genes coding LCE and KRT proteins induced a loss of differentiation of AK_L compared to AK_NL, which could lead to an increase in cell migration. In agreement, Zou et al. found that some keratin-family-related genes were strongly downregulated in AK and SCC compared to normal skin, whereas others were upregulated in SCC. *KRT16*, a type I intermediate filament cytoskeletal protein, was upregulated in AK_L. *KRT16* is weakly expressed in normal skin, but its expression is induced in skin pathologies leading to the activation of innate immunity [32].

In addition, the NFκB1 transcription factor was identified as an upstream regulator of genes upregulated in both comparisons SCC vs. H and AK_L vs. AK_NL. NFκB1 is known to play an important role in the regulation of the immune response and tumorigenesis [33]. As already reported, NFκB dysregulation contributes to SCC progression [34]. Loercher et al. showed that in vitro, this transcription factor modulates the expression of more than 60% of genes differently expressed between SCC cells and healthy keratinocytes [34]. This role was confirmed in vivo by Poligone et al., who showed that increased NFκB activity led to AKs development. Interestingly in their study, the authors suggest that NFκB activation selectively triggers AKs that do not self-resolve [35].

Taken together, our data show that deregulated genes in AK_L are related to functions very similar to those that are also deregulated in SCCs. Therefore, these lesions should be treated in the same way as SCCs.

An increasing number of studies describe the role of noncoding RNAs (ncRNAs) in SCC carcinogenesis, such as miRNA [15], lncRNA, or circular RNAs [20]. It would be interesting to complete our study with the expression data of these ncRNA between both AK classes.

Nevertheless, we identified one molecule, tivozanib, which is a VEGFR inhibitor and a PDGFR/KIT inhibitor presenting an inverse signature of AK_L lesions, suggesting that this molecule could reverse the AK_L profile to the AK_NL profile. Tivozanib is used to treat adults with metastatic renal cell carcinoma [36,37].

To our knowledge, no therapy inhibiting VEGFR has been tested for the treatment of SCCs or AKs. Indeed, the activation of the VEGF pathway in the development of SCCs has not really been highlighted. However, a rather old study suggested that VEGF is critical in the development of nonmelanoma skin cancers, as UV radiation can induce VEGF production by keratinocytes [38]. Using a murine multistep chemical carcinogenesis model of squamous cell carcinoma, Alitalo et al. demonstrated that VEGF-C/VEGF-D-blocking agents inhibit inflammatory skin carcinogenesis. This treatment reduced SCC development by decreasing macrophage infiltration [39]. Our work, as well as others, shows the role of the immune system in SCC progression [40]. So, compounds that selectively inhibit VEGF pathways and reduce inflammation could be promising therapies for SCCs.

## 4. Materials and Methods

### 4.1. Human Tissue Samples

In most cases, SCC surgical resection was performed by resecting a spindle-shaped skin sample (Figure S6). Such a spindle is made of three types of tissue:

1. The carcinoma (referred to in this study as the lesion (L) skin site) itself represented as a disk on the scheme; although SCCs may often display a round shape, their shape may vary and sometimes be random;
2. The safety margins (referred to in this study as the peri-lesion (PL) skin site) are based on clinical and healthcare system recommendations. The margins are adapted to the cancer histological class (5 mm for an SCC, 3 mm for a nodular type of basal cell carcinoma, etc.) but also to the risk of recurrence of the cancer (e.g., higher for a trabecular basal cell carcinoma than for a nodular).
3. The spindle tips (on each side of the carcinoma, referred to in this study as the non-lesion (NL) skin site) that exist in clinically considered healthy skin removed in order to allow for a regular suture from one side of the spindle to the other one.

On specific skin sites, such as the vertex and legs, round-shaped (not spindle-shaped) skin samples were surgically removed. In such cases, a skin graft was needed; healthy tissue was sampled from the graft taken from the neck. For each skin sample, the anatomical site from which it was sampled is mentioned using a dedicated letter (Table S8).

Right before resecting the surgical spindle, a 2 mm diameter punch (yellow disks in Figure S6a,b corresponding to the dark spots in Figure S6c) was used to sample skin from the 3 skin types: L, PL and NL. The skin samples were taken from the center of the SCC. When a thick keratin layer (i.e., parakeratosis) was present, the keratin layer was removed after anesthesia to avoid causing the patient pain. The thick keratin layer was removed because it does not contain any cells and transcriptomic analysis required cells from which RNA can be extracted for further transcriptomic analysis.

Two histological cuts (dots in Figure S6a) were made on the fixed skin sample: first along a lesion diameter crossing the L and PL punch sites and second on the spindle edge in order to obtain access to a diagnosis corresponding to the NL punch sites. The histological sections (7 µm tick) were stained with hematoxylin and eosin (H&E). The punches were visible on the histological sections (green arrows in Figure S6d): this ensures that the transcriptomic analysis was performed on skin samples from known histological classes.

For the current study, only punches collected on skin samples corresponding to the following histological classes validated by two independent pathologists were included: invasive or in situ SCCs (all pooled together, as we only had less than 10% of in situ SCCs), AKs of any stage (I, II or III) and healthy (H) skin. Figures S1 and S2 display characteristic pictures of H and SCC histological classes.

A total of 72 tissue samples, including 23 SCC, 21 AK and 28 H, skin punched either from the NL sites (24), L sites (23), or PL sites (4) were isolated from 24 patients (Table S9 and Figure S7). All human tissues were obtained from patients who gave their informed consent to be enrolled into the SpectroLive study (NCT0295626) that was approved by the French National Drug Agency (ANSM) and by the ethical committee (CPP Est III). Two patients were included several times in the study: patient 18 is the same person as patients 26 and 45; patient 35 is the same person as patient 41.

### 4.2. mRNA Extraction and Integrity

Total RNA was purified with an E.Z.N.A.^®^ HP Total RNA Kit (Omega Bio-Tek). RNA integrity was assessed using the Agilent 2100 Bioanalyzer System and RNA 6000 Nano kit (Agilent Technologies, Santa Clara, CA, USA). Only RNA with an RNA Integrity Number >7 were used for microarray experiments.

### 4.3. Microarray Analysis

A total of 100 ng of extracted mRNAs from patients were used to perform the microarray experiment. Genome-wide expression profiling was performed using the low-input QuickAmp labeling kit and human SurePrint G3 8x60K pangenomic microarrays (Agilent Technologies, Santa Clara, CA, USA). The gene expression data were processed using Feature Extraction (Agilent Technologies). The data discussed in this publication have been deposited in NCBI’s Gene Expression Omnibus and are accessible on 15 March 2023 through GEO Series accession number GSE207744 (https://www.ncbi.nlm.nih.gov/geo/query/acc.cgi?acc=GSE207744 (accessed on 25 January 2023)).

### 4.4. Samples Grouping

The NL and L tissue samples were divided into two groups. The reference group included samples from 14 patients presenting at least one L and one NL sample. These patients were used to establish the lesional signature (L vs. NL). The second group of samples, called the test group, was used to determine the optimized method for the generation of the DEGs list (Table S1).

The “AK” group will be later classified using the analysis results obtained for the «NL» and «L» reference subjects.

### 4.5. Differentially Expressed Genes

Two lists of DEGs were produced. The first was generated by a LIMMA (R) analysis pipeline and the second using GeneSpring GX software v11.5 (Agilent Technology, Santa Clara, CA, USA).

For the first list of DEGs, raw data. txt files in the Agilent format were loaded in R (version 4.0.2 (22 June 2020)). The «NL» and «L» reference subjects were used for a differential expression analysis using the Limma package (version 3.46) from BioConductor (10.1214/16-AOAS920). NL corresponds to the H histological class, and L corresponds to the SCC histological class.

After a background correction (with an offset value = 50) and a normalization between arrays using the quantile method, the linear models were fitted using both the diagnosis and patient index. A t statistic was computed and a *p*-value adjustment for multiple testing (*p*-value = 0.05) was made using the Benjamini–Hochberg with a minimum of log2 fold change = 2.

For the second list of DEGs, the raw data were downloaded in GeneSpring GX software v12 (Agilent Technology). The data were normalized using the shift to the 75th percentile method. The « NL » and « L » reference subjects were used for a differential expression analysis using a t-test (with a Bonferroni FWER correction) with a *p*-value of <0.05 and an absolute fold change (|FC|) of >2.

A schematic view of the two methods for obtaining the lists of DEGs is shown in Figure 1.

### 4.6. Principal Components Analysis (PCA) and Home-Made Classification

Both lists of DEGs were used in the two PCAs. Only the reference NL group and reference L group were used to compute the PCAs (Table S1). The number of principal components to keep was fixed by a cumulative explained variance >75%.

Then, on both generated PCAs, the test sample patient group was added to the plot using their coordinates in the PCA space. We then computed the reference NL group centroid coordinates and the reference L group centroid coordinates by averaging the coordinates of the samples from each reference group. For each tested sample patient, the Euclidean distance to each centroid was computed on the PCA’s two first component plans. Each test sample was labeled according to the closest centroid. We then compared the labeled with the histological known classification of the test samples to determine which differential analysis method between LIMMA and GeneSpring gave us the most reliable DEGs list to identify the histological class of a sample. The most accurate DEGs and PCA were then used to produce the homemade classification of the AK samples. The classification was performed using the nearest centroid to define the AK subclass. Thus two groups of AK, “AK_NL” and “AK_L” were defined.

### 4.7. Hierarchical Clustering

The hierarchical clustering analysis was conducted using Cluster 3.0 and TreeView 1.6 (Java Treeview) using uncentered correlation and complete linkage.

### 4.8. Functional Analysis

The gene annotation was based on gene ontology, and the enrichment for specific biological functions was determined using the FuncAssociate 2.0 program (FuncAssociate: The Gene Set Functionator) [41]. Top upstream regulators (FDR < 0.1) and top pathways enrichment (FDR < 0.01) were highlighted using ShinyGo [42].

### 4.9. Gene Set Enrichment Analysis

The gene set enrichment analysis (GSEA) was used to check whether an a priori defined set of genes showed statistically significant concordant differences between two biological states. The GSEA was performed using the Java tool developed at the Broad Institute (Cambridge, MA, USA). Unsupervised GSEA was conducted with the whole C2 collection of curated gene sets from the molecular signatures database (MSigDB). The enrichment score (ES) was determined after 1000 permutations, as previously described [43,44].

### 4.10. Connectivity Map

The clue.io platform (https://clue.io/query accessed on 23 June 2022) was used to compare the AK progression signature (AK_L vs. AK_NL) to the collection of hundreds of thousands of L1000 gene expression profiles from cells exposed to reference perturbagens, available in the public LINCS Connectivity Map (CMap) resource [45]. Perturbagens were identified based on the CMap connectivity score (tau). Only perturbagens with a ǀTauǀ of 90 or higher were selected in our study.

### 4.11. Statistical Analysis

Results are expressed as means ± standard deviation (SD). Data were analyzed using Graph Pad Prism software (Version 8.0.2; GraphPad Prism, San Diego, CA, USA). The significance between the four groups: NL, AK_NL, AK_L and L was evaluated by ANOVA followed by a Tukey’s post-hoc test for multiple comparisons. To determine genes significantly deregulated between AK_L and AK_NL conditions a t-test with a *p*-value < 0.05 and a |FC| > 2 was used.

## 5. Conclusions

For the first time, AK lesions were stratified based on their transcriptomic profile. Our data propose new diagnostics and therapeutics targets of AKs that would progress into SCCs and will contribute to optimizing patient management. A clinical trial including a six-month follow-up of patients will have to be performed to identify the effective progression risk of the two subclasses identified in the present study.

## Figures and Tables

**Table 1:** Histological and molecular classifications of the AK patient samples. Color gradient (Green-Yellow-Red) highlighting distances from the samples to the centroid. The green color corresponds to the longest distance and the red color to the shortest distance AK, actinic keratosis.

Patient Inclusion Number	Sample Name	Histopathological Diagnostic (Grade)	Transcriptomic Profile		
			DistToNL	DistToL	AK Classification
1	001_C_AK	AK_I	6.58	19.58	AK_NL
6	006_A_AK	AK_II	25.09	3.19	AK_L
16	016_B_AK	AK_I	6.60	19.09	AK_NL
17	017_A_AK	AK_I	2.62	25.89	AK_NL
28	028_F_AK	AK_II	28.06	8.21	AK_L
29	029_C_AK	AK_II	3.22	21.54	AK_NL
35 = 41	041_A_AK	AK_III	4.73	23.30	AK_NL
48	048_F_AK	AK_I	5.37	19.38	AK_NL
56	056_B_AK	AK_I	17.39	7.48	AK_L
75	075_U_AK	AK_I	3.87	21.06	AK_NL
77	077_B_AK_1	AK_I	4.37	20.82	AK_NL
	077_B_AK_2	AK_I	11.55	13.42	AK_NL
82	082_al_AK	AK_III	18.73	8.62	AK_L
	082_B_AK_1	AK_I	13.85	11.14	AK_L
	082_B_AK_2	AK_I	13.87	11.08	AK_L
90	090_A_AK	AK_I	5.98	19.69	AK_NL
109	109_C’_AK_1	AK_I	0.26	24.91	AK_NL
	109_C’_AK_2	AK_II	13.58	11.31	AK_L
115	115_F_AK	AK_II	18.38	6.72	AK_L
119	119_C_AK_1	AK_I	14.99	11.23	AK_L
	119_C_AK_2	AK_I	10.21	16.26	AK_NL

## Data Availability

The data discussed in this publication have been deposited in NCBI’s Gene Expression Omnibus (GEO) and are accessible through GEO Series accession number GSE207744 (https://www.ncbi.nlm.nih.gov/geo/query/acc.cgi?acc=GSE207744, accessed on 15 March 2023). The following secure token has been created to allow review of record GSE207744 while it remains in private status: qradaagsvhoxxob.

## Supplementary Materials

The following supporting information can be downloaded at: https://www.mdpi.com/article/10.3390/ijms24065937/s1.

## References

[1] Green A.C., Soyer H.P., Prow T.W., Jemec G.B.E. (2015). Epidemiology of Actinic Keratoses. Current Problems in Dermatology.

[2] Warino L., Tusa M., Camacho F., Teuschler H., Fleischer A.B., Feldman S.R. (2006). Frequency and cost of actinic keratosis treatment. Dermatol. Surg..

[3] Skobowiat C., Dowdy J.C., Sayre R.M., Tuckey R.C., Slominski A. (2011). Cutaneous hypothalamic-pituitary-adrenal axis homolog: Regulation by ultraviolet radiation. Am. J. Physiol. Endocrinol. Metab..

[4] Holman C.D., Armstrong B.K., Evans P.R., Lumsden G.J., Dallimore K.J., Meehan C.J., Beagley J., Gibson I.M. (1984). Relationship of solar keratosis and history of skin cancer to objective measures of actinic skin damage. Br. J. Dermatol..

[5] Frost C.A., Green A.C., Williams G.M. (1998). The prevalence and determinants of solar keratoses at a subtropical latitude (Queensland, Australia). Br. J. Dermatol..

[6] Criscione V.D., Weinstock M.A., Naylor M.F., Luque C., Eide M.J., Bingham S.F., for the Department of Veteran Affairs Topical Tretinoin Chemoprevention Trial Group (2009). Actinic keratoses: Natural history and risk of malignant transformation in the Veterans Affairs Topical Tretinoin Chemoprevention Trial. Cancer.

[7] Glogau R.G. (2000). The risk of progression to invasive disease. J. Am. Acad. Dermatol..

[8] Werner R.N., Stockfleth E., Connolly S.M., Correia O., Erdmann R., Foley P., Gupta A.K., Jacobs A., Kerl H., Lim H.W. (2015). Evidence- and consensus-based (S3) Guidelines for the Treatment of Actinic Keratosis—International League of Dermatological Societies in cooperation with the European Dermatology Forum—Short version. J. Eur. Acad. Dermatol. Venereol..

[9] Caddick J., Green L., Stephenson J., Spyrou G. (2012). The psycho-social impact of facial skin cancers. J. Plast. Reconstr. Aesthetic Surg..

[10] Röwert-Huber J., Patel M.J., Forschner T., Ulrich C., Eberle J., Kerl H., Sterry W., Stockfleth E. (2007). Actinic keratosis is an early in situ squamous cell carcinoma: A proposal for reclassification. Br. J. Dermatol..

[11] Righi V., Reggiani C., Tarentini E., Mucci A., Paganelli A., Cesinaro A.M., Mataca E., Kaleci S., Ferrari B., Meleti M. (2021). Metabolomic Analysis of Actinic Keratosis and SCC Suggests a Grade-Independent Model of Squamous Cancerization. Cancers.

[12] Fernández-Figueras M.T., Carrato C., Sáenz X., Puig L., Musulen E., Ferrándiz C., Ariza A. (2015). Actinic keratosis with atypical basal cells (AK I) is the most common lesion associated with invasive squamous cell carcinoma of the skin. J. Eur. Acad. Dermatol. Venereol..

[13] Schmitz L., Kahl P., Majores M., Bierhoff E., Stockfleth E., Dirschka T. (2016). Actinic keratosis: Correlation between clinical and histological classification systems. J. Eur. Acad. Dermatol. Venereol..

[14] Hameetman L., Commandeur S., Bavinck J.N.B., Wisgerhof H.C., de Gruijl F.R., Willemze R., Mullenders L., Tensen C.P., Vrieling H. (2013). Molecular profiling of cutaneous squamous cell carcinomas and actinic keratoses from organ transplant recipients. BMC Cancer.

[15] Chitsazzadeh V., Coarfa C., Drummond J.A., Nguyen T., Joseph A., Chilukuri S., Charpiot E., Adelmann C.H., Ching G., Nguyen T.N. (2016). Cross-species identification of genomic drivers of squamous cell carcinoma development across preneoplastic intermediates. Nat. Commun..

[16] Lambert S.R., Mladkova N., Gulati A., Hamoudi R., Purdie K., Cerio R., Leigh I., Proby C., Harwood C.A. (2014). Key differences identified between actinic keratosis and cutaneous squamous cell carcinoma by transcriptome profiling. Br. J. Cancer.

[17] Nguyen T.N., Rajapakshe K., Nicholas C., Tordesillas L., Ehli E.A., Davis C.M., Coarfa C., Flores E.R., Dickinson S.E., Curiel-Lewandrowski C. (2020). Integrative transcriptomic analysis for linking acute stress responses to squamous cell carcinoma development. Sci. Rep..

[18] Queen D., Shen Y., Trager M.H., Lopez A.T., Samie F.H., Lewin J.M., Niedt G.W., Geskin L.J., Liu L. (2020). UV biomarker genes for classification and risk stratification of cutaneous actinic keratoses and squamous cell carcinoma subtypes. FASEB J..

[19] Chen H., Yang J., Wu W. (2021). Seven key hub genes identified by gene co-expression network in cutaneous squamous cell carcinoma. BMC Cancer.

[20] Das Mahapatra K., Pasquali L., Søndergaard J.N., Lapins J., Nemeth I.B., Baltás E., Kemény L., Homey B., Moldovan L.-I., Kjems J. (2020). A comprehensive analysis of coding and non-coding transcriptomic changes in cutaneous squamous cell carcinoma. Sci. Rep..

[21] Hudson L.G., Gale J.M., Padilla R.S., Pickett G., Alexander B.E., Wang J., Kusewitt D.F. (2010). Microarray analysis of cutaneous squamous cell carcinomas reveals enhanced expression of epidermal differentiation complex genes. Mol. Carcinog..

[22] Zou D.-D., Xu D., Deng Y.-Y., Wu W.-J., Zhang J., Huang L., He L. (2021). Identification of key genes in cutaneous squamous cell carcinoma: A transcriptome sequencing and bioinformatics profiling study. Ann. Transl. Med..

[23] Yan G., Li L., Zhu S., Wu Y., Liu Y., Zhu L., Zhao Z., Wu F., Jia N., Liao C. (2021). Single-cell transcriptomic analysis reveals the critical molecular pattern of UV-induced cutaneous squamous cell carcinoma. Cell Death Dis..

[24] Campione E., Di Prete M., Di Raimondo C., Costanza G., Palumbo V., Garofalo V., Mazzilli S., Franceschini C., Dika E., Bianchi L. (2022). Topical Treatment of Actinic Keratosis and Metalloproteinase Expression: A Clinico-Pathological Retrospective Study. IJMS.

[25] Zhang W., Huang X., Huang R., Zhu H., Ye P., Lin X., Zhang S., Wu M., Jiang F. (2022). MMP1 Overexpression Promotes Cancer Progression and Associates with Poor Outcome in Head and Neck Carcinoma. Comput. Math. Methods Med..

[26] Mitsui H., Suárez-Fariñas M., Gulati N., Shah K.R., Cannizzaro M.V., Coats I., Felsen D., Krueger J.G., Carucci J.A. (2014). Gene Expression Profiling of the Leading Edge of Cutaneous Squamous Cell Carcinoma: IL-24-Driven MMP-7. J. Investig. Dermatol..

[27] Nindl I., Dang C., Forschner T., Kuban R.J., Meyer T., Sterry W., Stockfleth E. (2006). Identification of differentially expressed genes in cutaneous squamous cell carcinoma by microarray expression profiling. Mol. Cancer.

[28] Zheng L.-Q., Wang R., Chi S.-M., Li C.-X. (2019). Matrix metalloproteinase 1: A better biomarker for squamous cell carcinoma by multiple microarray analyses. G. Ital. Dermatol. Venereol..

[29] Dang C., Gottschling M., Roewert J., Forschner T., Stockfleth E., Nindl I. (2006). Tenascin-C patterns and splice variants in actinic keratosis and cutaneous squamous cell carcinoma. Br. J. Dermatol..

[30] Azin M., Demehri S. (2021). Innate Lymphoid Cells: New Targets for Cutaneous Squamous Cell Carcinoma Immunotherapy. J. Investig. Dermatol..

[31] Steeb T., Petzold A., Hornung A., Wessely A., Berking C., Heppt M.V. (2022). Spontaneous regression rates of actinic keratosis: A systematic review and pooled analysis of randomized controlled trials. Sci. Rep..

[32] Lessard J.C., Piña-Paz S., Rotty J.D., Hickerson R.P., Kaspar R.L., Balmain A., Coulombe P.A. (2013). Keratin 16 regulates innate immunity in response to epidermal barrier breach. Proc. Natl. Acad. Sci. USA.

[33] Yu H., Lin L., Zhang Z., Zhang H., Hu H. (2020). Targeting NF-κB pathway for the therapy of diseases: Mechanism and clinical study. Signal Transduct. Target. Ther..

[34] Loercher A., Lee T.L., Ricker J.L., Howard A., Geoghegen J., Chen Z., Sunwoo J.B., Sitcheran R., Chuang E.Y., Mitchell J.B. (2004). Nuclear factor-kappaB is an important modulator of the altered gene expression profile and malignant phenotype in squamous cell carcinoma. Cancer Res..

[35] Poligone B., Hayden M.S., Chen L., Pentland A.P., Jimi E., Ghosh S. (2013). A Role for NF-κB Activity in Skin Hyperplasia and the Development of Keratoacanthomata in Mice. PLoS ONE.

[36] Rini B.I., Pal S.K., Escudier B.J., Atkins M.B., Hutson T.E., Porta C., Verzoni E., Needle M.N., McDermott D.F. (2020). Tivozanib versus sorafenib in patients with advanced renal cell carcinoma (TIVO-3): A phase 3, multicentre, randomised, controlled, open-label study. Lancet Oncol..

[37] Chang E., Weinstock C., Zhang L., Fiero M.H., Zhao M., Zahalka E., Ricks T.K., Fourie Zirkelbach J., Qiu J., Yu J. (2022). FDA Approval Summary: Tivozanib for Relapsed or Refractory Renal Cell Carcinoma. Clin. Cancer Res..

[38] Brauchle M., Funk J.O., Kind P., Werner S. (1996). Ultraviolet B and H2O2 Are Potent Inducers of Vascular Endothelial Growth Factor Expression in Cultured Keratinocytes. J. Biol. Chem..

[39] Alitalo A.K., Proulx S.T., Karaman S., Aebischer D., Martino S., Jost M., Schneider N., Bry M., Detmar M. (2013). VEGF-C and VEGF-D Blockade Inhibits Inflammatory Skin Carcinogenesis. Cancer Res..

[40] Bottomley M.J., Thomson J., Harwood C., Leigh I. (2019). The Role of the Immune System in Cutaneous Squamous Cell Carcinoma. Int. J. Mol. Sci..

[41] Berriz G.F., Beaver J.E., Cenik C., Tasan M., Roth F.P. (2009). Next generation software for functional trend analysis. Bioinformatics.

[42] Ge S.X., Jung D., Yao R. (2020). ShinyGO: A graphical gene-set enrichment tool for animals and plants. Bioinformatics.

[43] Mootha V.K., Lindgren C.M., Eriksson K.-F., Subramanian A., Sihag S., Lehar J., Puigserver P., Carlsson E., Ridderstråle M., Laurila E. (2003). PGC-1α-responsive genes involved in oxidative phosphorylation are coordinately downregulated in human diabetes. Nat. Genet..

[44] Subramanian A., Tamayo P., Mootha V.K., Mukherjee S., Ebert B.L., Gillette M.A., Paulovich A., Pomeroy S.L., Golub T.R., Lander E.S. (2005). Gene set enrichment analysis: A knowledge-based approach for interpreting genome-wide expression profiles. Proc. Natl. Acad. Sci. USA.

[45] Subramanian A., Narayan R., Corsello S.M., Peck D.D., Natoli T.E., Lu X., Gould J., Davis J.F., Tubelli A.A., Asiedu J.K. (2017). A Next Generation Connectivity Map: L1000 Platform and the First 1,000,000 Profiles. Cell.

