# Supplementary material for: Transcriptomic Study on Human Skin Samples: Identification of Two Subclasses of Actinic Keratoses
Source: Int J Mol Sci. 2023 Mar 21;24(6):5937. doi: 10.3390/ijms24065937 (PMC10058209; doi:10.3390/ijms24065937)
Supplement: Supplementary file 1 [file ijms-24-05937-s001.zip › Figure S1.pptx]

## Slide 1
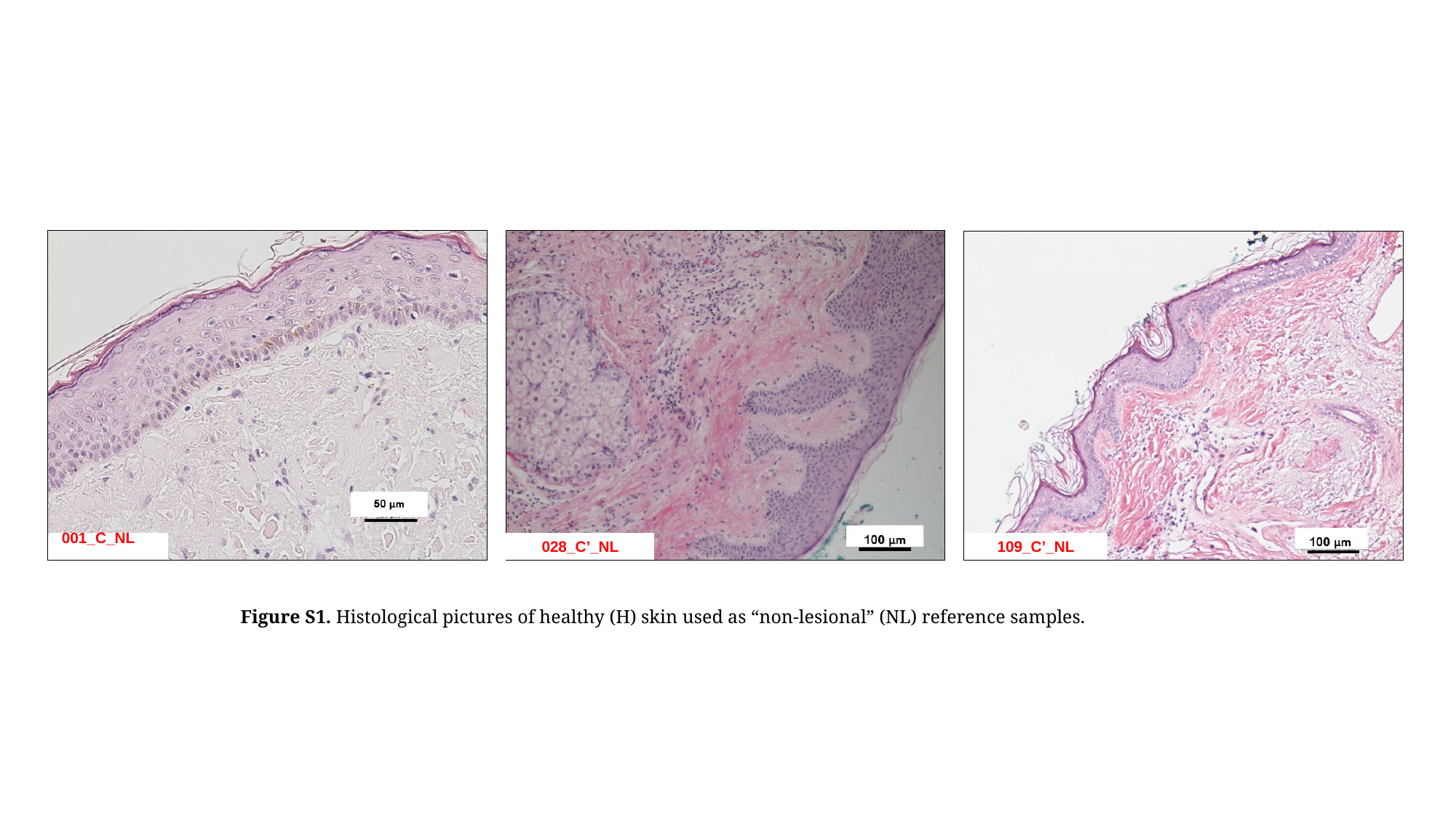

001_C_NL
028_C’_NL
109_C’_NL
Figure S1. Histological pictures of healthy (H) skin used as “non-lesional” (NL) reference samples.
