# Supplementary material for: Transcriptomic Study on Human Skin Samples: Identification of Two Subclasses of Actinic Keratoses
Source: Int J Mol Sci. 2023 Mar 21;24(6):5937. doi: 10.3390/ijms24065937 (PMC10058209; doi:10.3390/ijms24065937)
Supplement: Supplementary file 1 [file ijms-24-05937-s001.zip › Figure S3.pptx]

## Slide 1
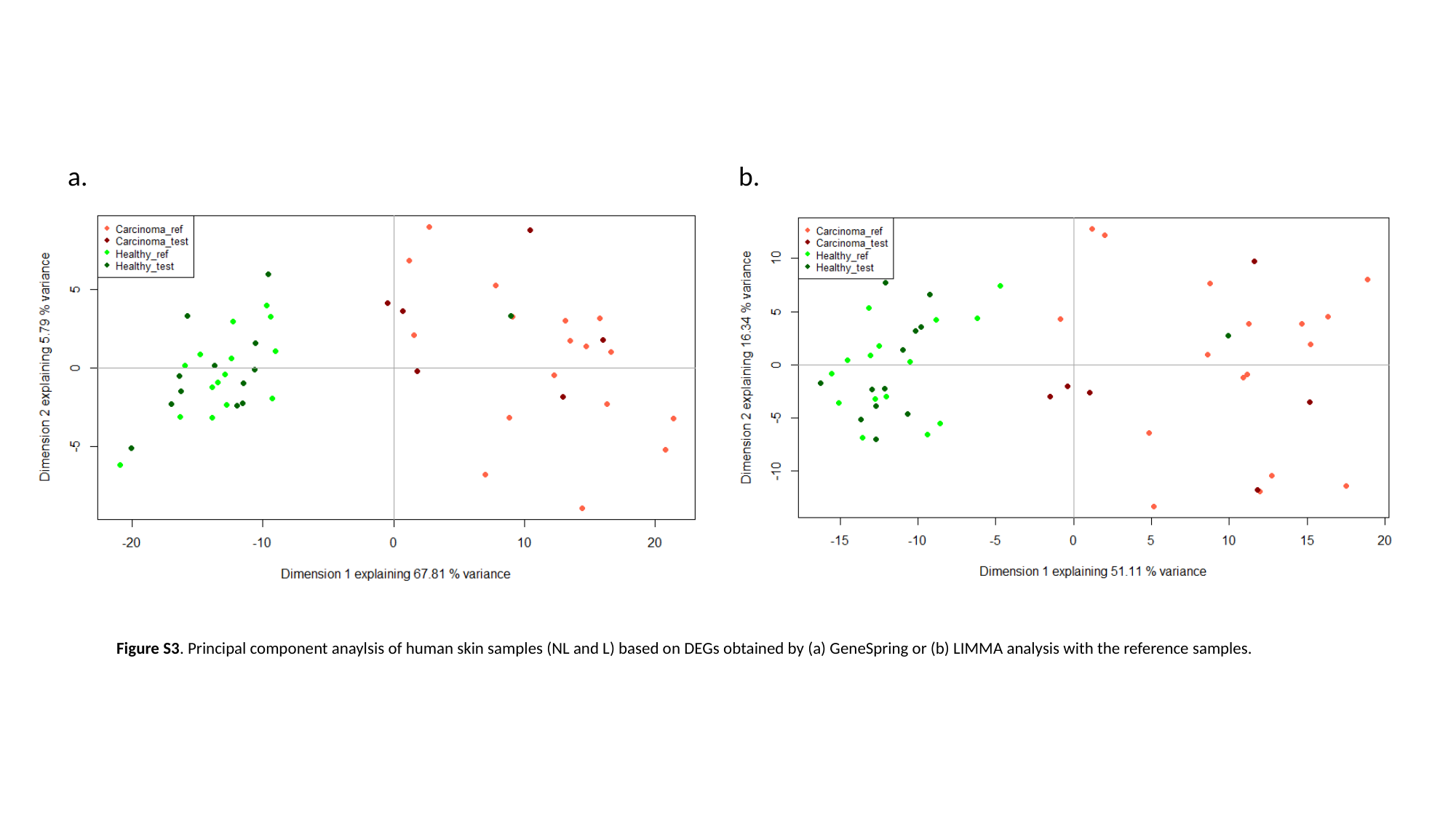

a.
b.
Figure S3. Principal component anaylsis of human skin samples (NL and L) based on DEGs obtained by (a) GeneSpring or (b) LIMMA analysis with the reference samples.
