# Supplementary material for: Transcriptomic Study on Human Skin Samples: Identification of Two Subclasses of Actinic Keratoses
Source: Int J Mol Sci. 2023 Mar 21;24(6):5937. doi: 10.3390/ijms24065937 (PMC10058209; doi:10.3390/ijms24065937)
Supplement: Supplementary file 1 [file ijms-24-05937-s001.zip › Figure S4.pptx]

## Slide 1
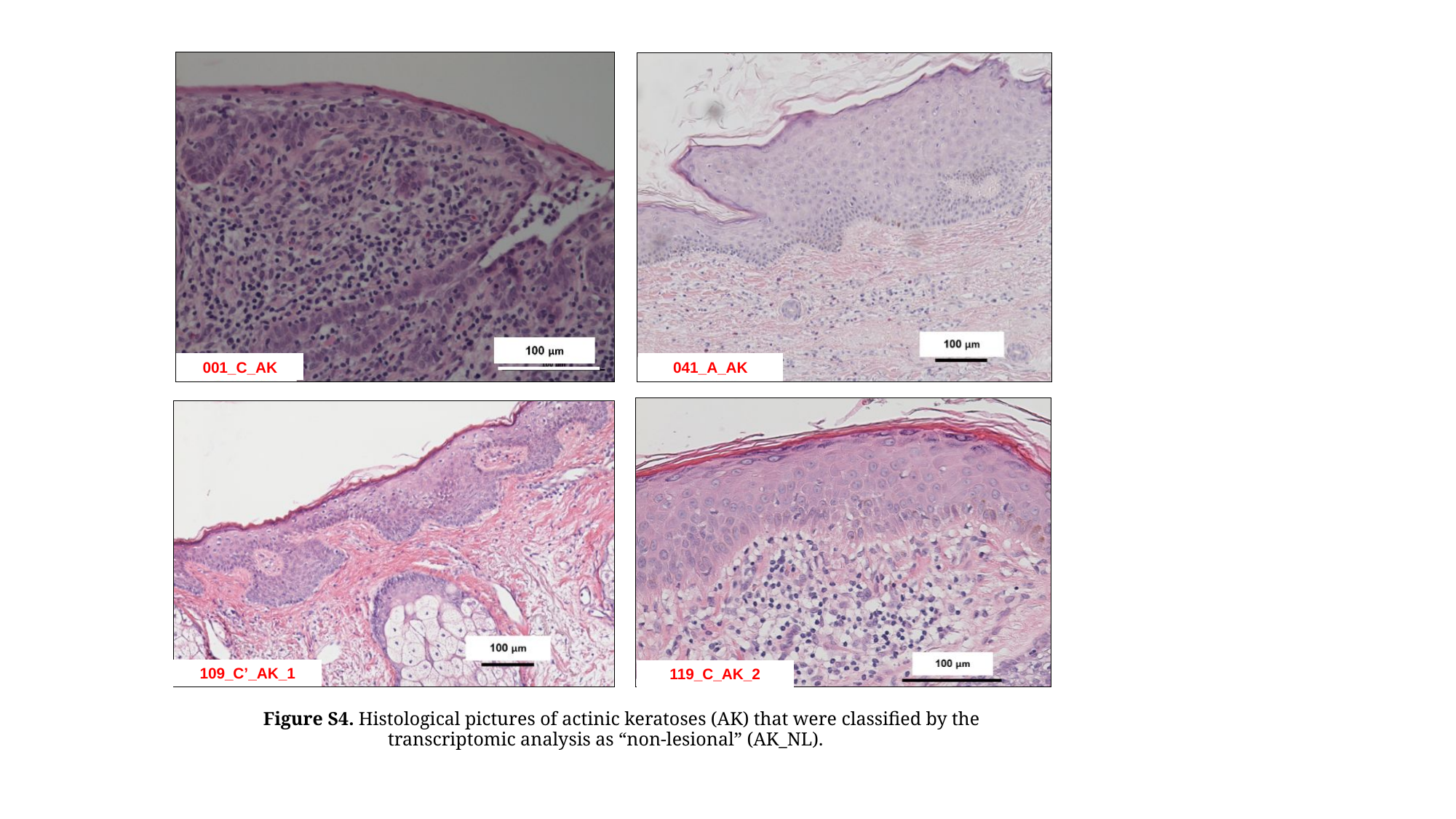

001_C_AK
041_A_AK
109_C’_AK_1
119_C_AK_2
Figure S4. Histological pictures of actinic keratoses (AK) that were classified by the transcriptomic analysis as “non-lesional” (AK_NL).
