# Supplementary material for: Transcriptomic Study on Human Skin Samples: Identification of Two Subclasses of Actinic Keratoses
Source: Int J Mol Sci. 2023 Mar 21;24(6):5937. doi: 10.3390/ijms24065937 (PMC10058209; doi:10.3390/ijms24065937)
Supplement: Supplementary file 1 [file ijms-24-05937-s001.zip › Figure S5.pptx]

## Slide 1
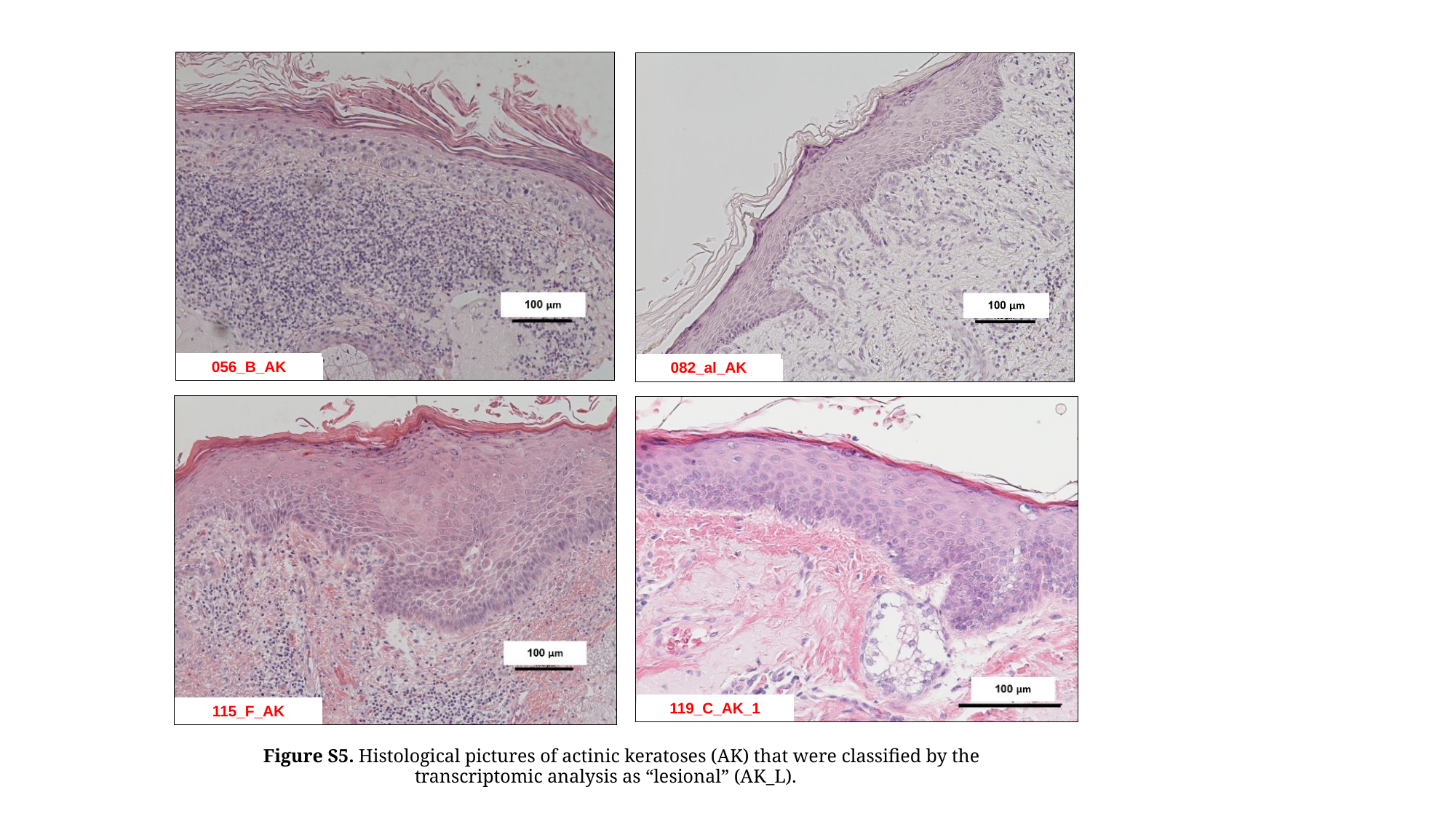

056_B_AK
082_al_AK
119_C_AK_1
115_F_AK
Figure S5. Histological pictures of actinic keratoses (AK) that were classified by the transcriptomic analysis as “lesional” (AK_L).
