# Supplementary material for: Transcriptomic Study on Human Skin Samples: Identification of Two Subclasses of Actinic Keratoses
Source: Int J Mol Sci. 2023 Mar 21;24(6):5937. doi: 10.3390/ijms24065937 (PMC10058209; doi:10.3390/ijms24065937)
Supplement: Supplementary file 1 [file ijms-24-05937-s001.zip › Figure S6.pptx]

## Slide 1
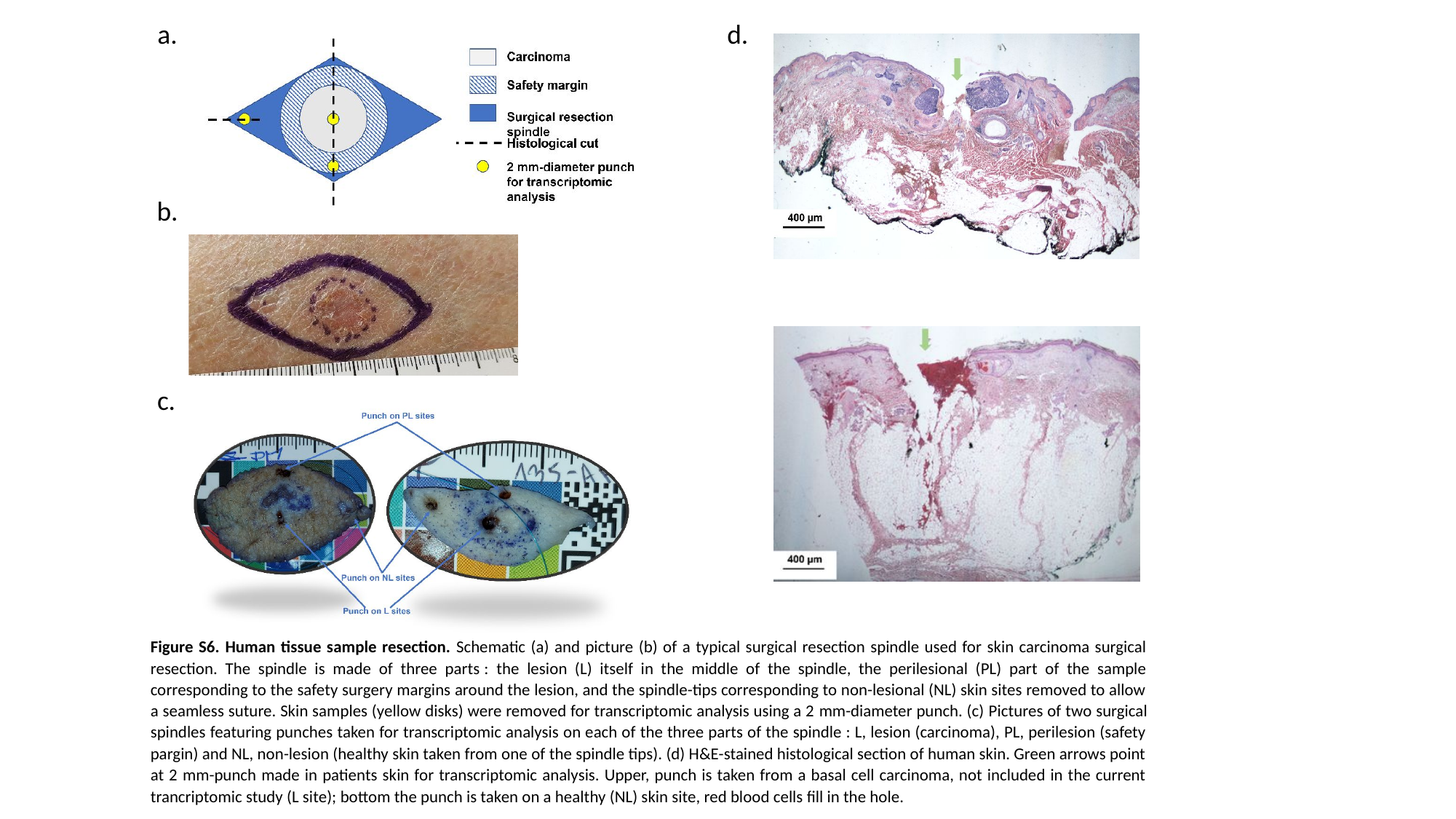

a.
d.
b.
c.
Figure S6. Human tissue sample resection. Schematic (a) and picture (b) of a typical surgical resection spindle used for skin carcinoma surgical resection. The spindle is made of three parts : the lesion (L) itself in the middle of the spindle, the perilesional (PL) part of the sample corresponding to the safety surgery margins around the lesion, and the spindle-tips corresponding to non-lesional (NL) skin sites removed to allow a seamless suture. Skin samples (yellow disks) were removed for transcriptomic analysis using a 2 mm-diameter punch. (c) Pictures of two surgical spindles featuring punches taken for transcriptomic analysis on each of the three parts of the spindle : L, lesion (carcinoma), PL, perilesion (safety pargin) and NL, non-lesion (healthy skin taken from one of the spindle tips). (d) H&E-stained histological section of human skin. Green arrows point at 2 mm-punch made in patients skin for transcriptomic analysis. Upper, punch is taken from a basal cell carcinoma, not included in the current trancriptomic study (L site); bottom the punch is taken on a healthy (NL) skin site, red blood cells fill in the hole.
