# Supplementary material for: Transcriptomic Study on Human Skin Samples: Identification of Two Subclasses of Actinic Keratoses
Source: Int J Mol Sci. 2023 Mar 21;24(6):5937. doi: 10.3390/ijms24065937 (PMC10058209; doi:10.3390/ijms24065937)
Supplement: Supplementary file 1 [file ijms-24-05937-s001.zip › Figure S7.pptx]

## Slide 1
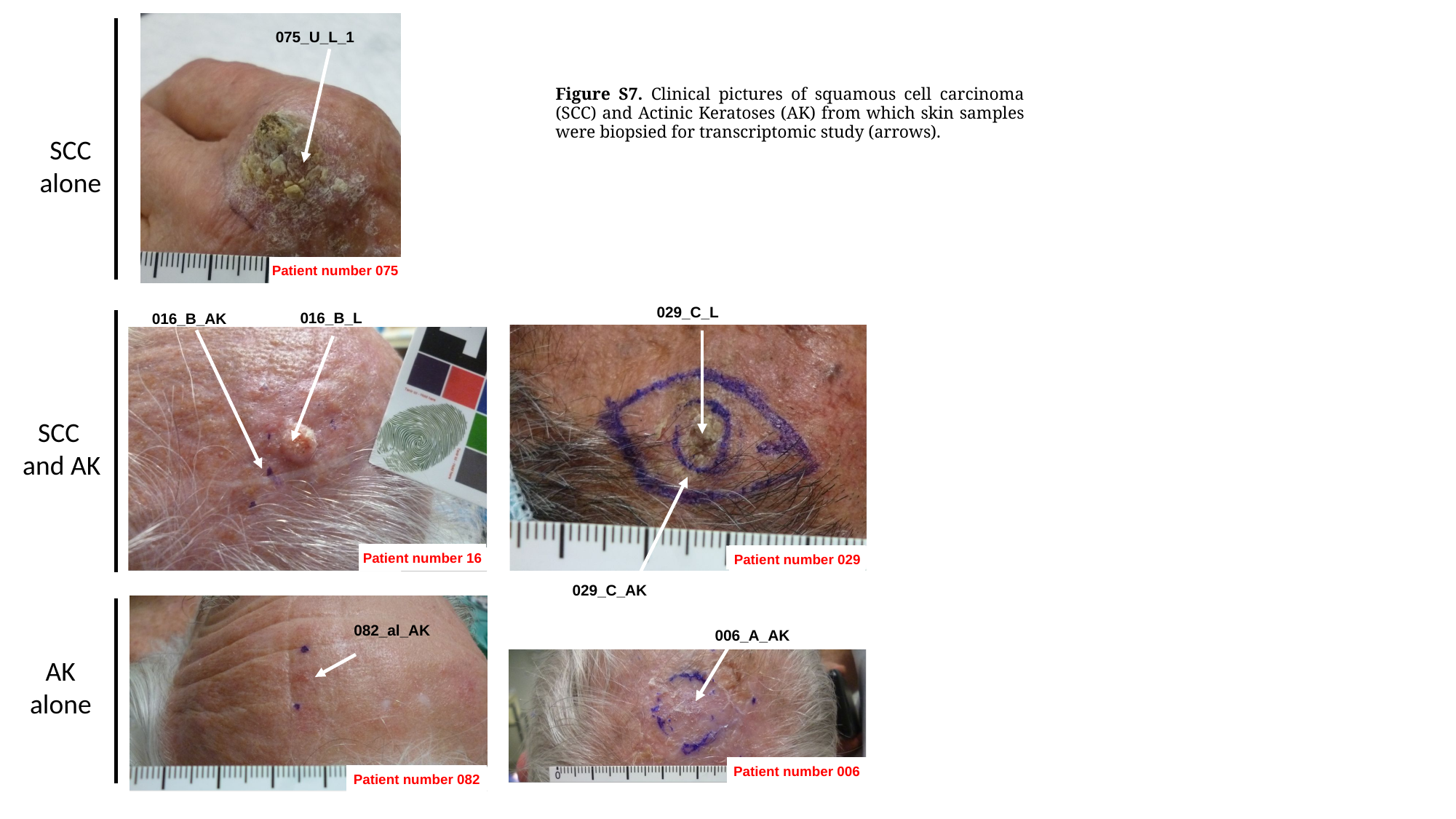

075_U_L_1
Figure S7. Clinical pictures of squamous cell carcinoma (SCC) and Actinic Keratoses (AK) from which skin samples were biopsied for transcriptomic study (arrows).
SCC
alone
Patient number 075
029_C_L
016_B_AK
016_B_L
SCC
and AK
Patient number 16
Patient number 029
029_C_AK
082_al_AK
006_A_AK
AK
alone
Patient number 006
Patient number 082
